# Glycolysis and glycolytic enzymes in acute myeloid leukemia: Warburg and beyond

**DOI:** 10.3389/fphar.2026.1823211

**Published:** 2026-05-20

**Authors:** Kristina Seiler, Yasmeen H. Mady, Bruce E. Torbett, Jean-Emmanuel Sarry, Mario P. Tschan

**Affiliations:** 1 Institute of Tissue Medicine and Pathology, University of Bern, Bern, Switzerland; 2 Graduate School of Cellular and Biomedical Sciences, University of Bern, Bern, Switzerland; 3 Department of Pediatrics, School of Medicine, University of Washington, Seattle, WA, United States; 4 Department of Clinical Pathology, South Egypt Cancer Institute, Assiut University, Assiut, Egypt; 5 Center for Immunity and Immunotherapies, Seattle Children’s Research Institute, Seattle, WA, United States; 6 Institute for Stem Cell and Regenerative Medicine, Seattle, WA, United States; 7 Université de Toulouse, Centre de Recherches en Cancérologie de Toulouse, Inserm, CNRS, Toulouse, France; 8 Centre Hospitalier Universitaire de Toulouse, Toulouse, France

**Keywords:** AML, glycolysis, metabolic inhibitors, metabolic reprogramming, non-canonical functions, OxPhos, Warburg effect

## Abstract

The ability to generate and regulate energy, maintain metabolic flux, and preserve redox homeostasis is the basis of all cellular processes in both healthy and diseased tissues. While the importance of glycolytic metabolism in cancer biology is well established, the clinical translation of metabolic targeting strategies remains limited. A deeper understanding of how energy flux is regulated and how metabolic enzymes contribute to cell survival, proliferation, differentiation, and therapy response will help identify cancer-specific metabolic dependencies and drive novel therapeutic approaches. Recent advances in molecular and metabolic profiling have highlighted the multifaceted roles of glycolytic enzymes beyond their canonical functions. In this review, we summarize current knowledge on glycolytic enzymes and their involvement in normal and malignant myelopoiesis, with a particular focus on acute myeloid leukemia (AML). AML cells exhibit high glycolytic activity and frequently overexpress key glycolytic enzymes. Beyond their metabolic functions, these enzymes also exert regulatory roles in signaling, transcriptional control, and redox balance. Here, we discuss both the canonical and non-canonical functions of glycolytic enzymes and evaluate their potential as therapeutic targets in AML.

## 1 Introduction

In the era of precision medicine, impressive progress has been made in identifying and targeting specific oncogenic drivers. These drivers are often part of large signaling networks and cancers, sadly, frequently evolve to develop resistance to targeted therapies. Furthermore, targeted therapies are highly specific, and their success is largely dependent on the individual molecular characteristics of a patient’s cancer. In the search for broader anti-tumor strategies, targeting cellular energy pathways has emerged as a central focus of the scientific community and is currently being pursued through extensive preclinical and clinical investigations. Metabolic pathways as potential targets have two distinct advantages: firstly, they are common to various cancers and secondly, some cancer cells have been shown to be dependent on certain metabolic pathways that these targets serve. To date, there has been little clinical success with exploiting this approach given the targets are often necessary for normal tissue function, limiting the therapeutic window [reviewed in (Kim, 2019; Stuani et al., 2019)]. However, tumor-specific metabolic targets and dependencies are being identified. A more complete understanding of cellular metabolic regulation will advance the efficiency of targeting strategies. Furthermore, it is becoming evident that many metabolic enzymes possess functions outside of their known role in metabolism. These activities can be surprisingly broad and involve crucial processes such as regulation of gene expression and cell cycle progression. The purpose of this review is to highlight glycolytic features in healthy *versus* cancerous tissues, focus on glycolytic enzymes and explore their canonical and non-canonical roles in metabolism, proliferation, development, and drug resistance in one of most therapy-resistant aggressive cancers, acute myeloid leukemia (AML).

## 2 Metabolic reprogramming in hematopoiesis and AML

### 2.1 The Warburg effect: a feature of normal and malignant hematopoietic cell proliferation

Roughly a century ago, Otto Warburg discovered that cancer cells display a distinct energy metabolism (Warburg, 1926). His observation revealed that tumor cells are glycolytically very active and ferment glucose via pyruvate into lactate even in the presence of oxygen. In contrast, healthy tissues preferentially fully oxidize glucose through oxidative phosphorylation (OxPHOS), netting more ATP *per* molecule of glucose as compared to fermentation. This metabolic feature of malignancy has been termed aerobic glycolysis and has been recognized as a hallmark of cancer in 2011 (Hanahan and Weinberg, 2011). In healthy cells, the production of lactate is normally kept at a minimum and fermentation primarily occurs under anaerobic conditions. Warburg initially suspected that cancer cells might have dysfunctional mitochondria (Warburg, 1956), but this has been proven to be incorrect and it has been established that oxidative metabolism is not compromised in most cancer cells (Moreno-Sánchez et al., 2007). While a cancer cell’s energy metabolism looks different compared to differentiated tissue, certain metabolic features may be a result of the malignancy’s proliferative state rather than cancer specific. For example, activated T-cells were shown to also perform aerobic glycolysis and ferment glucose to lactate despite not being in a hypoxic environment (Hume et al., 1978; Fox et al., 2005).

### 2.2 ATP is only part of the story

**FIGURE 1:**
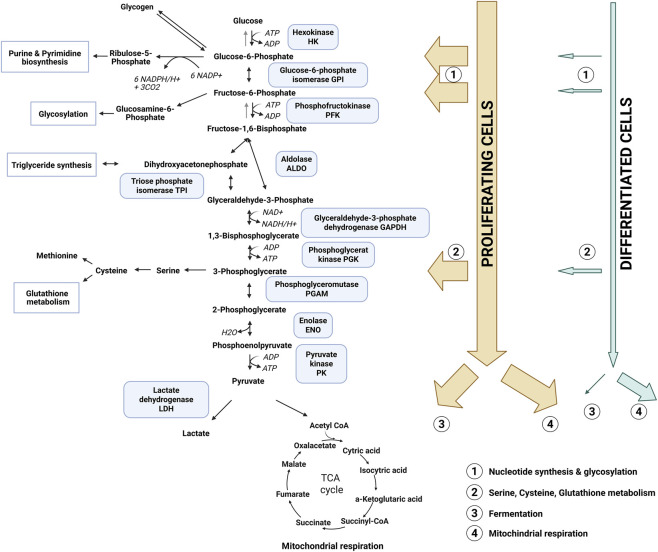
Overview of glycolysis and its enzymes. Schematic representation of the glycolytic pathway and associated enzymes, illustrating the distinct metabolic fates of glucose in proliferating and differentiated/resting cells. In proliferating cells, glycolytic intermediates are extensively diverted into anabolic pathways to support biomass production, including nucleotide synthesis, glycosylation, and lipid biosynthesis, as well as the generation of reducing equivalents. In contrast, differentiated or resting cells primarily metabolize glucose to pyruvate, which is subsequently shuttled to mitochondria for oxidative phosphorylation. Only a minor fraction of glucose (approximately 10%) is redirected into biosynthetic pathways (1, 2), and fermentation (3) is minimal under these conditions.

In terms of ATP production, oxidation is far more efficient as compared to lactic acid fermentation as it yields 18-times more ATP *per* molecule of oxidized glucose (Campbell, 1993). At first glance, fermentation appears to be a wasteful metabolic pathway, begging the question as to why proliferating cells or cancer cells would prefer such an inefficient energy strategy. The first study generating findings supporting cancer cell use of aerobic glycolysis was made in 1993 by Guppy et al. (Guppy et al., 1993). The authors showed that the rate at which ATP is being produced by aerobic glycolysis can indeed exceed OxPHOS in conditions of unlimited glucose supply. Secondly and potentially equally important, glucose metabolism also supplies cells with essential building blocks for various anabolic pathways. For example, glucose-6-phosphate (G6P), the product of the first step in glycolysis, can contribute to three different pathways (Figure 1). Apart from classical glycolysis, G6P can feed into the pentose phosphate pathway (PPP) or converted to glycogen for energy storage. The PPP generates reducing equivalents (NADPH) to thwart oxidative stress as well as pentoses for synthesis of nucleotides and aromatic amino acids. If G6P continues within classical glycolysis, it is interconverted into fructose-6-phosphate (F6P), which, apart from further glycolytic metabolization, can support the hexosamine pathway for generation of glycoproteins (Marshall et al., 1991). Halfway through the glycolytic pathway the intermediate 3-phosphoglycerate (3 PG) is generated, which can feed into *de novo* serine-methionine biosynthesis, sustaining nucleotide, sphingolipid and glutathione formation (Bridgers, 1965). Considering the large requirements for biomass during proliferation, it becomes evident that complete oxidation of glucose fails to supply macromolecular precursors for various biosynthetic building blocks such as nucleotides and fatty or amino acids. Importantly, most of the acetyl-CoA, which serves as the carbon donor in fatty acid synthesis, is derived from glucose [and to some extent from glutamine) (reviewed in (Röhrig and Schulze, 2016)]. Anabolic metabolism of proliferating cells is essential for three biological processes: bioenergetics, biosynthetic pathways and redox balance [reviewed in (Cantor and Sabatini, 2012)]. While resting cells can cover their demands mainly by catabolic metabolism of fatty acids, amino acids and glucose via OxPHOS, proliferating cells are met with increased anabolic demands. That aerobic glycolysis supplies cellular building blocks, rather than solely generating ATP, was strengthened when Guppy et al. demonstrated that breast cancer cells use OxPHOS to generate the majority of the tumor cellular ATP (roughly 80%) (Guppy et al., 2002). More recent studies in lung tumors using radiolabeled glucose identified enhanced glycolytic oxidation through the TCA cycle in highly proliferative regions, again highlighting that glucose does get metabolized through the oxidative pathway in glycolytic cancers (Hensley et al., 2016). These data were collected from flash frozen tissue sections of patients who had received ^13^C labeled glucose infusions before surgery, elegantly capturing the actual metabolic activity *in vivo*. Discrepancies between metabolic flux in *ex vivo* culture systems due to different nutrient availability compared to *in vivo* situations is a major concern in metabolic research and will be addressed separately in this review. Taken together, upregulation of glycolytic activity in tumor tissue, and potentially other proliferative tissues, is currently thought to serve the increase in biosynthetic demand and support redox balance. It is highly likely that under proliferative stress, tumor cells manage to upregulate both glycolysis and OxPHOS to sustain the increase in biomass and energy requirements.

### 2.3 Glucose competition and microenvironment acidification

Another interesting aspect of upregulated glucose consumption was brought up by two separate studies, which concluded that it serves as a defense mechanism against immune cells. Within the tumor microenvironment, cancer cells and anti-tumor immune cells compete for glucose and by upregulating glucose uptake, cancer cells may try to outpace and deprive the immune cells of their necessary glucose supply (Chang C.-H. et al., 2015; Ho et al., 2015). Along the same line, a study by Sonveaux et al. highlighted that while tumors preferentially metabolize glucose into lactate instead of fully oxidizing the glucose, lactate in turn is afterwards taken up again by neighboring cancer cells and prominently fuels oxidative metabolism, as it can be oxidized into pyruvate again (Sonveaux et al., 2008). Furthermore, studies have shown that in glucose-deprived environments, cancer cells can adapt by upregulating monocarboxylate transporters (MCTs), particularly MCT1, to import extracellular lactate. This adaptation enables cancer cells to use lactate as an alternative fuel, maintaining their energy production and promoting survival under nutrient-limited conditions (Almeida et al., 2024). While this allows symbiotic shuttling of nutrients between different tumor areas, it also acidifies the tumor microenvironment, possibly impairing functions of invading T-cells (Fischer et al., 2007) as well as monocytes and dendritic cells (Gottfried et al., 2006).

## 3 The metabolic landscape of AML is complex

AML is a heterogeneous group of myeloid cancers originating from distinct myeloid stem and progenitor cells at various stages of development. AML cells are generally thought to rely heavily on glycolysis, with studies demonstrating that their glucose consumption can be up to 20-fold higher than that of normal hematopoietic cells. This increased glycolytic activity has been evidenced by significantly elevated glucose uptake in AML cells within the bone marrow microenvironment, as visualized by 18-fluoro-2-deoxyglucose (FDG) imaging (Ye et al., 2018; Deng et al., 2023). This glycolytic dependence is further supported by the activation of signaling pathways such as Akt/mTOR, which are known to enhance glycolytic flux and promote cell survival, particularly under conditions of increased glucose availability (Chen et al., 2023). Additionally, AML cells exhibit significant metabolic plasticity, enabling adaptation to therapeutic pressure. Catalano et al. demonstrated that AML cells preferentially rely on long-chain fatty acids as a primary energy source; however, when fatty acid oxidation is impaired, they readily shift toward increased glucose or glutamine utilization to sustain energetic demands (Catalano et al., 2023).

**FIGURE 2:**
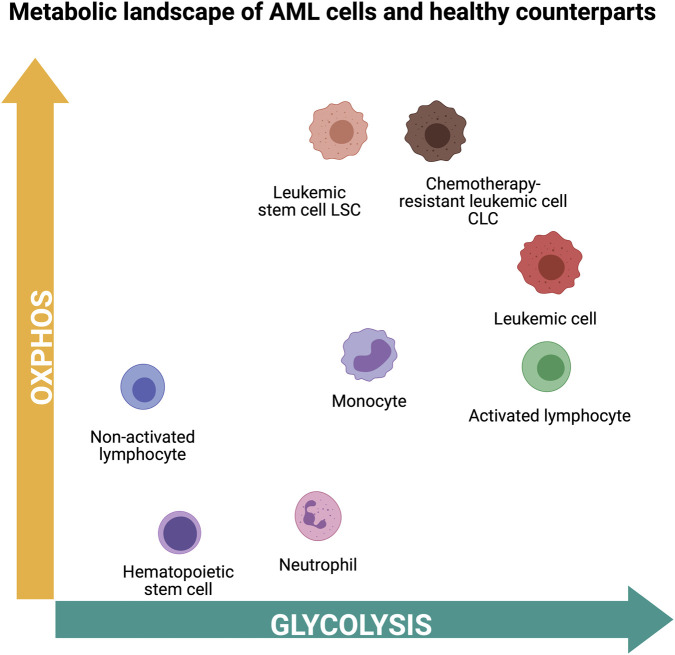
Metabolic positioning of healthy hematopoietic and myeloid leukemic cell populations along the glycolysis-oxidative phosphorylation axis. Schematic representation of the relative metabolic states of hematopoietic progenitor and immune cells, as well as myeloid leukemic populations, along a continuum from oxidative phosphorylation (OxPHOS) to glycolysis. Quiescent cells, including hematopoietic stem cells and non-activated lymphocytes, primarily rely on mitochondrial metabolism. Upon activation, immune cells such as lymphocytes and monocytes increase glycolytic flux to support proliferation and effector functions. Acute myeloid leukemic cells display metabolic heterogeneity. Bulk leukemic cells are typically more glycolytic, whereas leukemic stem cells (LSCs) and chemotherapy-resistant leukemic cells (CLCs) are more reliant on OxPHOS. Neutrophils represent a distinct population with a strong dependence on glycolysis. This schematic highlights the diversity and plasticity of metabolic states across healthy and malignant hematopoietic cell types.

A unique feature of AML metabolism is its systemic effect on glucose homeostasis. AML cells induce insulin resistance in normal tissues, ensuring high glucose availability for leukemic cells whose uptake is insulin-independent (Ye et al., 2018). Just as the metabolic activity is diverse among healthy blood cells at different stages of differentiation and depending on their activation status, subpopulations with distinct metabolic features can be identified in AML as well (Figure 2). Leukemic stem cells (LSCs), often representing the apex of clonal malignant development, rely heavily on OxPHOS despite maintaining low reactive oxygen species (ROS) levels. This dependency is supported by increased mitochondrial mass and reliance on mitophagy to maintain ROS homeostasis (Culp-Hill et al., 2021; Egan and Schimmer, 2023). Disruption of OxPHOS through BCL-2 family inhibition further highlights this vulnerability: treatment with the BCL-2/BCL-xL inhibitor Navitoclax increased ROS levels and selectively induced LSC death while sparing healthy CD34^+^ cells (Konopleva et al., 2006; Lagadinou et al., 2013). Building on these findings, the more selective BCL-2 inhibitor, venetoclax, was shown to impair mitochondrial respiration and tricarboxylic acid (TCA) cycle activity, independently of its canonical BCL-2 inhibition mechanism, leading to increased ROS and cell death in LSCs, while sparing healthy CD34^+^ cells (Roca-Portoles et al., 2020). The importance of mitochondria and TCA cycle in AML is further highlighted by the advancement of mitochondrial metabolism inhibitor CPI-613 (Devimistat) into clinical trials (Pardee et al., 2014; Pardee et al., 2018). CPI-613 inhibits the mitochondrial enzymes pyruvate dehydrogenase (PDH) and the α-ketoglutarate dehydrogenase complex (KDH), thereby impairing both glucose- and glutamine-fueled respiration and efficiently inducing cell death *in vitro*. However, clinical trials did not demonstrate a significant improvement in patient outcomes (Zachar et al., 2011; Pardee et al., 2014; Reddy et al., 2022). Although devimistat demonstrated promising results in phase I and II trials, it failed to show a significant therapeutic benefit in the phase III ARMADA 2000 clinical trial, which evaluated its combination with high-dose cytarabine and mitoxantrone in elderly patients with refractory or relapsed AML (Pardee et al., 2024).

Mutational backgrounds further modulate metabolic dependencies in AML. Mutations in isocitrate dehydrogenase (IDH1/2), present in approximately 20% of AML cases, lead to neomorphic activity and production of the oncometabolite 2-hydroxyglutarate (2-HG), which disrupts α-ketoglutarate (α-KG)-dependent dioxygenases, affecting epigenetic regulation and hematopoietic differentiation (Dang et al., 2009; Xu et al., 2011). From an energy metabolism perspective, IDH mutations enhance OxPHOS and mitochondrial respiration. IDH inhibitors fail to reduce this elevated mitochondrial activity, and oxidative metabolism contributes to resistance to these agents. However, combining IDH with OxPHOS inhibitors has demonstrated improved efficacy, highlighting the critical role of mitochondrial metabolism in therapy resistance (Stuani et al., 2021).

Other frequently mutated genes in AML such as FLT3, K-RAS, N-RAS, KIT, DNMT3A, and TP53, also influence metabolic regulation. Both FLT3 mutations and oncogenic KRAS have been associated with increased glycolytic activity. FLT3-ITD mutations drive glycolytic reprogramming in AML cells through activation of PI3K/AKT/mTOR and STAT5 signaling pathways, leading to increased expression of glycolytic enzymes such as HK2 and enhanced glucose uptake and lactate production (Ju et al., 2017). This metabolic shift supports biosynthetic demands, including nucleotide synthesis. In line with this, a metabolomics study in pediatric AML reported elevated levels of nucleosides, such as guanine, hypoxanthine, and adenosine, in FLT3-ITD samples compared to FLT3-wt, which the authors interpreted as indicative of a highly proliferative state (Stockard et al., 2018). Importantly, glycolytic inhibition has been shown to sensitize FLT3-ITD AML cells to FLT3 inhibitors such as sorafenib, suggesting that targeting metabolic vulnerabilities may enhance therapeutic responses. RUNX1 mutations are also linked to increased glycolysis and reduced OxPHOS activity characterized by upregulation of pyruvate dehydrogenase kinase 1 (PDK1) (Erdem et al., 2022). Importantly, metabolic profiling of chemotherapy-resistant AML cells has revealed a high oxidative signature, highlighting the role of oxidative metabolism in therapy resistance (Farge et al., 2017).

Taken together, these findings underscore significant metabolic heterogeneity and plasticity within AML, both between AML subtypes as well as within a heterogeneous AML population. This intra-tumoral diversity may involve a “symbiotic coexistence” of glycolytic cells in hypoxic niches generating lactate, which oxidative tumor cells utilize as fuel (Nakajima and Van Houten, 2013). Exploiting these metabolic dependencies through combinatorial strategies targeting glycolysis, OxPHOS, and other metabolic pathways holds promise for overcoming therapeutic resistance and improving outcomes in AML patients.

### 3.1 Glycolytic metabolism in normal hematopoiesis and AML

Generally, hematopoietic stem cells (HSCs) are thought to rely predominantly on glycolytic metabolism. Within the bone marrow stem cell niche, oxygen concentration is low, and HSCs are mostly quiescent with reduced metabolic activity. HIF1α mediates the suppression of pyruvate dehydrogenase, thereby limiting the conversion of pyruvate to acetyl-CoA and oxidative metabolism (Takubo et al., 2013) [for a more detailed review of HSC metabolism we refer to (Ito and Ito, 2018; Morganti et al., 2022)]. As HSCs start to cycle, their mitochondrial membrane potential increases, ATP generation surges and intracellular ROS rises. Once differentiated, metabolic activity has been shown to be associated with certain lineage specific subtypes. Lipopolysaccharide (LPS) stimulated pro-inflammatory M1 macrophages, for example, upregulate glycolysis along with a decrease in oxygen consumption, mediated in part through increased mTOR signaling as well as decreased AMPK activity [reviewed here (Kelly and O’Neill, 2015)]. On the other hand, M2 polarized macrophages are characterized by enhanced OxPHOS as well as enhanced fatty acid oxidation (Vats et al., 2006; Huang et al., 2014). Neutrophils have been shown to rely heavily on glycolysis both at normoxia as well as in hypoxic conditions, and both when unstimulated as well as when stimulated with LPS (Sadiku et al., 2020). After stimulation with LPS, glycolytic activity gets further upregulated, leading to an increase in glycolytic intermediaries as well as substrates of the PPP. Through glycogen storage and active gluconeogenesis, neutrophils have been shown to maintain high intracellular energetic status in both oxygen deprived and glucose deprived conditions (Sadiku et al., 2020).

For other hematopoietic cells, glycolytic activity varies greatly between resting and activated cell types. Herst et al. have shown that the relative level of glycolytic metabolism *versus* OxPHOS in T-cells increases largely with activation (Herst et al., 2011). In resting T-cells, they found glycolytic contribution to be low (10%). Upon activation, cells shift towards a more glycolytic metabolism, increasing the relative activity of glycolysis to 63% in activated T-cells. Bone marrow cells also show considerable glycolytic activity (62%). While leukemic cells have been shown to have increased glucose consumption compared to normal hematopoietic cells (Ye et al., 2018), comparison of absolute glucose consumption and precise metabolic activity between AML and healthy hematopoietic cells likely varies considerably depending on the subtype and activation status of the healthy counterpart.

**FIGURE 3:**
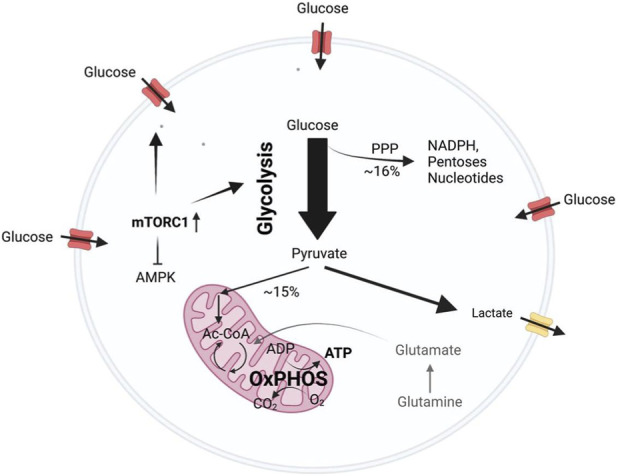
Glycolysis and energy metabolism in AML. Schematic overview of glucose metabolism in AML cells. High mammalian target of rapamycin complex 1 (mTORC1) activity, commonly observed in AML, promotes increased glycolytic flux. Approximately 16% of glucose-derived carbon is diverted into the pentose phosphate pathway (PPP) to support biosynthesis. The majority of pyruvate (∼85%) is converted to lactate, whereas a smaller fraction (∼15%) enters mitochondria to fuel oxidative phosphorylation (OxPHOS) and ATP production.

In healthy tissue, metabolic activity is usually tightly regulated. Glucose uptake as well as proliferation is generally directed via extracellular growth factor signaling and intracellular nutrient sensing kinases. Through oncogene activation, cancer cells manage to initiate nutrient uptake and proliferation largely autonomous and independent of extracellular signaling (reviewed in (Zhu and Thompson, 2019)). In AML, high glycolytic activity is driven by mTORC1 activity and high mTORC1 activity furthermore results in sensitivity to glycolysis inhibition (Poulain et al., 2017). Interestingly, a relatively large proportion of metabolized glucose feeds into the pentose phosphate pathway (16%), presumably supporting nucleotide synthesis and redox balance (Poulain et al., 2017) (Figure 3). While multiple *in vitro* studies have found high glycolytic activity to be associated with apoptosis resistance (Herst et al., 2011; Song et al., 2016), patient data suggests that highly glycolytic AML blasts are in fact more sensitive to therapy (Herst et al., 2011). High glycolytic activity in AML blast cells was found to be associated with longer first complete remission and increased overall survival when compared to blasts with only moderate glycolytic activity. A potential explanation offered by the authors is that highly glycolytic blasts may be less flexible in utilizing different energy-generating pathways resulting in increased sensitivity to metabolic stress. Interestingly, they also found that none of the three patient samples of which matched samples were obtained at diagnosis and after relapse, showed alteration in glycolytic activity between diagnosis and relapse (Herst et al., 2011). A comprehensive review exploring the relationship between glycolysis and chemoresistance in AML is available here (Yang et al., 2024).

### 3.2 Metabolic determinants of normal hematopoietic and myeloid differentiation

The importance of metabolic control of differentiation is highlighted by the fact that *ex vivo* propagation of HSCs remains a challenge but is so far most effective in an environment that limits O_2_ consumption and prevents excessive ROS production (Bai et al., 2019). Stem cell and progenitor populations with high ROS tend to enter cell cycle and differentiate. In order to limit flux of glycolytic metabolites into mitochondria, HSCs have been shown to suppress the conversion of pyruvate to acetyl-CoA through pyruvate dehydrogenase kinase mediated inhibition of pyruvate dehydrogenase (Takubo et al., 2013). There have also been reports implicating specific isoforms of glycolytic enzymes in the repopulation and differentiation ability of HSCs such as pyruvate kinase muscle isoform 2 (PKM2) and lactate dehydrogenase A (LDHA). Loss of either of these enzymes in mouse models impaired glycolytic activity and long-term bone marrow repopulation capacity under stress was compromised when HSCs of the respective knockout mice were serially transplanted (Wang et al., 2014). Furthermore, erythropoiesis was significantly impaired in LDHA^−/−^ mice (Wang et al., 2014), indicating the aerobic glycolytic dependency of erythroid lineage mostly for maintenance of cellular redox balance upon high oxygen exposure. Notably, mature erythrocytes lack mitochondria, underlining the importance of LDH to metabolize pyruvate.

Apart from glycolytic enzymes, mitochondrial metabolic pathways have also been implicated in HSC function. For instance, deletion of the TCA cycle enzyme fumarate hydratase (FH) impairs HSC self-renewal and compromises long-term repopulation capacity, highlighting the importance of intact mitochondrial metabolism for HSC maintenance (Guitart et al., 2017). While myeloid output is largely preserved under these conditions, lymphoid output is reduced, suggesting a differential sensitivity of hematopoietic lineages to disruptions in mitochondrial metabolism rather than a strict lineage-specific requirement. Similarly, Luchsinger et al. identified a subpopulation-specific role for Mitofusin 2 (Mfn2), a regulator of mitochondrial dynamics and ER-mitochondria interactions, where its loss selectively affected lymphoid repopulation without significantly altering the myeloid compartment. In contrast, increased glycolytic activity has been associated with myeloid-biased differentiation, as aged HSCs exhibit elevated glycolysis alongside a shift toward myeloid lineage output at the expense of lymphoid potential (Hennrich et al., 2018). Together, these findings point toward lineage-specific sensitivities to metabolic states; however, a strict segregation of oxidative phosphorylation and glycolysis between lymphoid and myeloid differentiation cannot be conclusively established.

### 3.3 Glycolytic enzymes in AML

**FIGURE 4:**
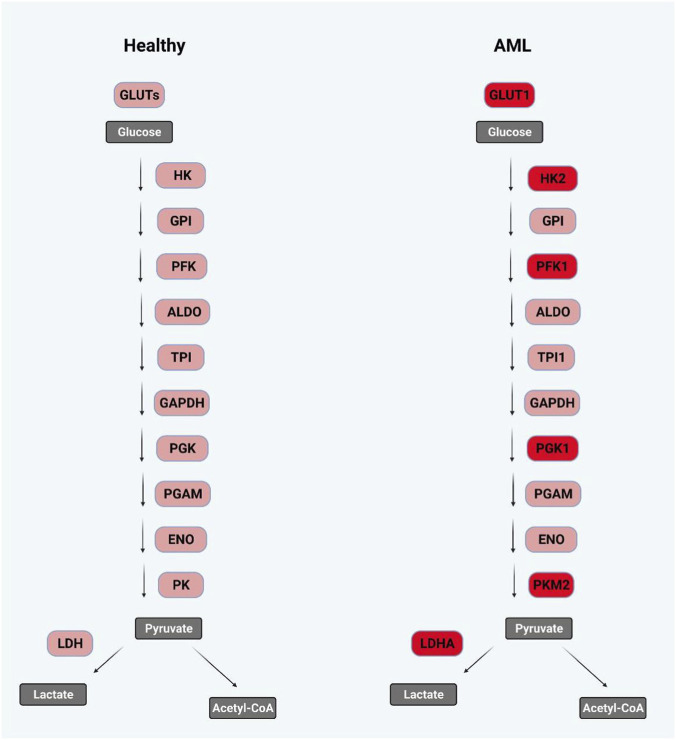
Glycolytic enzymes and isoforms implicated in AML. Schematic comparison of key glycolytic enzymes and isoforms expressed in healthy hematopoietic cells (left) versus AML cells (right). In AML, multiple components of the glycolytic pathway are upregulated or preferentially expressed as cancer-associated isoforms, including increased glucose uptake via GLUT1, enhanced activity of hexokinase 2 (HK2), phosphofructokinase-1 (PFK1), phosphoglycerate kinase 1 (PGK1), pyruvate kinase M2 (PKM2), and lactate dehydrogenase A (LDHA). These alterations collectively promote elevated glycolytic flux and lactate production, consistent with aerobic glycolysis. In contrast, healthy cells predominantly express alternative isoforms and exhibit more balanced metabolic routing of pyruvate toward mitochondrial oxidation. Beyond their canonical metabolic roles, several of these enzymes exert non-canonical functions in signaling, transcriptional regulation, and apoptosis, thereby contributing to leukemogenesis and therapy resistance. For detailed discussion of individual enzymes and their functional implications in AML, see main text.

Glycolytic enzymes catalyze the 10-step cytoplasmic process converting glucose to pyruvate, which can be further metabolized through either oxidative metabolism within the mitochondria or converted into lactate, catalyzed by LDH. A series of glycolytic enzymes have been associated with AML (summarized in Figure 4; Table 1). Stabilization of HIF1α and oncogenic MYC expression, as frequently seen in AML, increases the expression of various glycolytic enzymes such as hexokinase 2 (HK2), phosphofructokinase (PFK1), pyruvate kinase (PK) and LDHA, and promotes glucose import through induction of its transporter GLUT1 [reviewed in (Nagao et al., 2019)]. HK2, catalyzing the first step in glycolysis after glucose import, has been implicated in tumor growth and metastasis in various solid cancers and is thought to promote lactate production (Edry Botzer et al., 2016; Anderson et al., 2017). In AML, AKT-mediated HK2 upregulation has specifically been documented in the context of FLT3-ITD mutated samples, a molecular feature that is associated with poor prognosis (Ju et al., 2017). Both cell line models and AML patient samples positive for FLT3-ITD also showed greater therapeutic sensitivity to glycolytic inhibition compared to FLT3-wt samples (Ju et al., 2017). PFK1 catalyzes the conversion of fructose-6-phosphate to fructose-1,6-bisphosphate and has been reported to be overactive in AML, increasing glycolytic flux. This is achieved through increased availability of the allosteric activator of PFK1, fructose-2,6-bisphosphate, which in turn is generated via overexpression of phosphofructokinase-2/fructose-2,6-biphosphatase 3 (PFKFB3) (Feng and Wu, 2017). PFKFB enzymes are known regulators of glycolysis and increase glucose uptake and lactate production (Ros and Schulze, 2013). Overexpression of PGK1 has also been reported in AML patients, and association was found between high PGK1 expression and high risk AML, but not with a specific gene fusion or mutational profile (Gao et al., 2021). Accordingly, genetic silencing of PGK1 has led to decreased cellular viability in a cell line model of histiocytic lymphoma (U937) (Gao et al., 2021).

**TABLE 1:** Glycolytic isoforms overexpressed and/or associated with AML.

Enzyme/Isoform	Metabolic function	Role in cancer	Association
HK2	Glycolysis glucose -> glucose-6-phosphate	Overexpressed	FLT-mutation
PFK1	Glycolysis fructose-6-phosphate -> fructose-1,6-bisphosphate	Overactive	high mTOR activity
GAPDH	Glycolysis glyceraldehyde-3-phosphate -> glycerate-1,6-bisphosphate	Overexpressed	FAB-M4/5
PGK1	Glycolysis 1,3-diphosphoglycerate -> 3-phosphoglycerate	Overexpressed	High risk AML
PKM2	Glycolysis phosphoenolpyruvate -> pyruvate	Overexpressed	NPM1 mutation
LDHA	Lactate production pyruvate -> lactate	Prognostic value	AML
Associated pathways			
PFKFB3	Regulator of Glycolysis	Overexpressed	high mTOR activity
G6PD	Pentose Phosphate pathway	Overexpressed	AML with monocytic differentiation, NPM1c and DNMT3a mutation

In line with afore-mentioned increased PPP activity, levels of G6P dehydrogenase (G6PD) are markedly higher in AML cells compared to normal immature hematopoietic cells and high levels of G6PD have been found to correlate with adverse prognosis in the non-M3 AML patient TCGA dataset (Poulain et al., 2017). High G6PD expression was especially pronounced in AML with monocytic differentiation and was associated with NPM1c and DNMT3a mutations (Poulain et al., 2017). On the other hand, inhibition of certain glycolytic enzymes such as 6-phosphogluconate dehydrogenase (6PGD), PKM2 and LDHA has been shown to hamper leukemic growth (Wang et al., 2014; Bhanot et al., 2017; Poulain et al., 2017). In the case of PKM2, its deletion compromised leukemia initiation without affecting normal progenitor function (Wang et al., 2014). However, as discussed above, while normal homeostatic hematopoiesis was not affected in PKM2^−/−^ mice, serial transplantations showed that long-term repopulation capacity of hematopoietic progenitor cells was impaired, while the differentiation capacity of HSCs was largely spared (Wang et al., 2014). While often associated with cancer, PKM2 is interestingly also the predominant pyruvate kinase isoform in healthy HSCs (Wang et al., 2014). PKM2, in contrast to PKM1, can be dynamically regulated as it exists both in dimeric and tetrameric form which differ in their activity. In comparison, PKM1 forms stable tetramers with high substrate affinity, is constitutively active and thought to support oxidative pyruvate metabolization (Allen and Locasale, 2018). The dimeric form of PKM2 on the other hand is believed to drive aerobic glycolysis, while tetrameric PKM2 supports flux of pyruvate through OxPHOS [reviewed in (Wong et al., 2013)]. Even though the K*m* towards the substrate phosphoenolpyruvate (PEP) is lower for tetrameric PKM2 compared to dimers, PKM2 expression in proliferating cells with high substrate concentrations allows dynamic regulation of pyruvate allocation towards glycolytic and oxidative metabolism (Wong et al., 2013).

### 3.4 Alternative splicing and isoform expression of glycolytic enzymes

While some glycolytic protein isoforms are encoded by distinct genes, others arise from alternative splicing. Most genes typically express a predominant transcript, referred to as the most dominant transcript (MDT), which is conserved across healthy tissues (Gonzàlez-Porta et al., 2013; Ezkurdia et al., 2015). In cancer, including AML, alternative splicing is frequently dysregulated, leading to MDT switching and the expression of non-canonical isoforms (Sebestyén et al., 2015). This represents an additional layer of gene expression regulation beyond somatic mutations. Notably, aberrant splicing patterns are observed even in the absence of mutations in core splicing factors, indicating widespread alterations in RNA processing in AML (Anande et al., 2020).

A prominent example of how alternative splicing connects to glycolytic metabolism is the afore-mentioned PKM2, a splice variant of the PKM pre-mRNA, that is predominant in many types of cancer (Desai et al., 2013). Its expression is promoted by PTBP1, a regulator of exon inclusion, and is elevated in NPM1-mutated AML (Wang et al., 2019). In parallel, transcriptome-wide analyses in AML patient samples have identified recurrent alternative splicing events affecting genes such as FLT3 and NOTCH2. While these events are not directly linked to glycolytic regulation, they underscore the global extent of splicing dysregulation in AML.

Alternative splicing can also alter protein localization and function, as illustrated by α-enolase (ENO1), which encodes both a cytosolic glycolytic enzyme and a shorter nuclear isoform, c-Myc promoter-binding protein 1 (MBP1) (Subramanian and Miller, 2000). This functional diversification highlights how splicing can modulate metabolic enzyme activity beyond canonical glycolysis. Although mutations in splicing factors such as SF3B1, SRSF2, and ZRSR2 are recurrent in AML and are associated with adverse outcome (Yoshida et al., 2011; Papaemmanuil et al., 2016; Döhner et al., 2022), their direct involvement in regulating specific glycolytic splice variants, including ENO1, remains incompletely understood. Following initial toxicity challenges with early spliceosome inhibitors, newer compounds are currently being evaluated for their therapeutic potential in AML (Seiler et al., 2018).

These observations suggest that while individual splicing events can directly impact metabolic enzymes, the broader splicing dysregulation in AML likely extends beyond metabolism and remains insufficiently mapped.

## 4 Moonlighting functions of glycolytic enzymes

**FIGURE 5:**
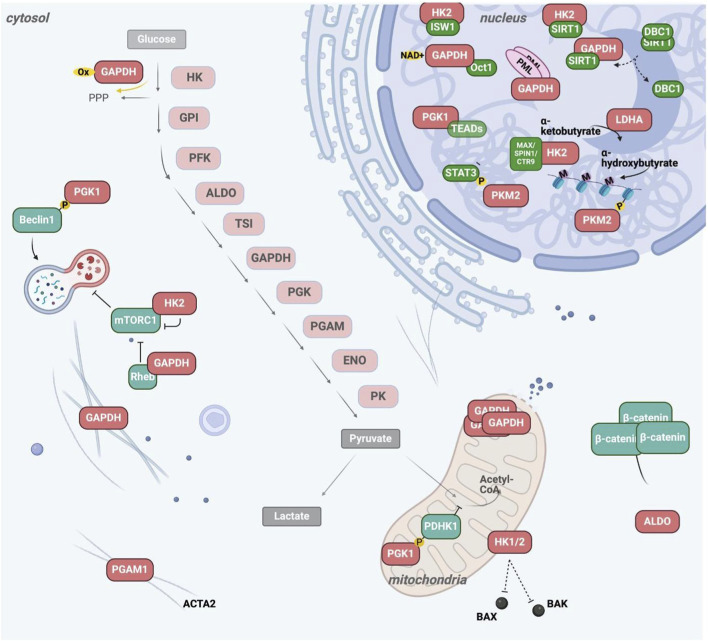
Non-canonical functions of glycolytic enzymes. Schematic overview of non-metabolic (“moonlighting”) functions of key glycolytic enzymes across cellular compartments. Hexokinases (HK1/2) localize to mitochondria, where they bind to voltage-dependent anion channels (VDAC) at the outer mitochondrial membrane, thereby preventing BAX/BAK-mediated apoptosis. In the cytosol, HK2 can interact with mTORC1, linking glycolysis to nutrient-sensing pathways. In the nucleus, HK2 associates with SIRT1 and chromatin regulators (e.g., MAX/SPIN1/CTR9 and ISW1), contributing to transcriptional control and DNA damage responses. Aldolase (ALDO) promotes activation of β-catenin signaling in the cytosol. Glyceraldehyde-3-phosphate dehydrogenase (GAPDH) exhibits multiple non-canonical functions, including nuclear interactions with PML bodies, transcription factor Oct1, and SIRT1, as well as release of the inhibitory factor DBC1. In the cytosol, GAPDH regulates metabolic flux by redirecting glucose into the pentose phosphate pathway (PPP) under oxidative conditions, inhibits mTORC1 via Rheb binding, and associates with microtubules. Under cellular stress, GAPDH can translocate to mitochondria and contribute to membrane permeabilization. Phosphoglycerate kinase (PGK1) participates in transcriptional regulation through interaction with TEAD transcriptional co-factors in the nucleus, phosphorylates Beclin1 to regulate autophagy in the cytosol, and modulates mitochondrial metabolism via phosphorylation of PDHK1. Phosphoglycerate mutase (PGAM1) interacts with cytoskeletal components such as α-smooth muscle actin (ACTA2). Lactate dehydrogenase A (LDHA) can localize to the nucleus, where it converts α-ketobutyrate to α-hydroxybutyrate, promoting histone H3K79 hypermethylation. These non-canonical functions link glycolytic enzymes to the regulation of apoptosis, transcription, chromatin organization, autophagy, and metabolic signaling, thereby modulating to tumor development and therapy responses.

Glycolytic enzymes are classically defined by their role in catalyzing the conversion of metabolic substrates into specific intermediates within the glycolytic pathway. However, accumulating evidence reveals a far more complex functional landscape. Beyond their canonical metabolic activities, several glycolytic enzymes exert additional, non-metabolic functions (Figure 5; reviewed in 84). While several of these non-canonical functions of glycolytic enzymes described below have been identified in diverse cellular systems and cancer types, we explicitly indicate where evidence derives from AML. Where direct evidence in AML is limited, findings from other systems are discussed for their potential relevance, while acknowledging that their applicability to AML remains to be established.

Glycolytic kinases normally phosphorylate metabolite substrates within the glycolytic pathway. There is now emerging evidence that their kinase activity is not limited to metabolic substrates but that they can indeed phosphorylate proteins as well, finetuning and regulating cellular homeostasis on various levels. A prominent example is PKM2, known for catalyzing the last step of glycolysis, converting phosphoenolpyruvate (PEP) and ADP into pyruvate and ATP. In non-hematopoietic cancer models, upon epidermal growth factor receptor (EGFR) and platelet-derived-growth factor receptor (PDGFR) signaling, PKM2 itself first gets phosphorylated by extracellular signal-regulated kinase (ERK) (Yang et al., 2012a), leading to a series of conformational changes. These changes expose a nuclear localization signal and nuclear translocation is promoted (Yang et al., 2012b). Inside the nucleus, PKM2 then uses PEP to act as a protein kinase and phosphorylate histone H3, which promotes HDAC3 removal, histone H3 K9-acetylation and gene expression. The regions targeted for PKM2 mediated histone modifications are, for example, the promoter of the *MYC gene*, whose upregulation promotes glycolytic gene expression and supports tumor growth (Yang et al., 2012a; 2012b). In T cells, PKM2 has been shown to phosphorylate and activate STAT3 upon SLC5A12 mediated lactate uptake, again acting as a protein kinase (Pucino et al., 2019). The authors show that STAT3 activation results in increased fatty acid synthesis and IL17 production, highlighting regulation of cellular metabolism through moonlighting activity of glycolytic enzymes. Not only phosphorylation but also other protein modifications have been reported to regulate activity and spatial expression patterns of PKM2. In the case of AML, SUMOylation of PKM2 at the lysine residue K270 confers an oncogenic role via triggering nuclear translocation and subsequent promoting of the degradation of myeloid differentiation transcription factor RUNX1 (Xia et al., 2021).

Another metabolic enzyme, albeit outside of the immediate glycolytic pathway, with known protein kinase functions is phosphoglycerate kinase 1 (PGK1). In glioblastoma and other cancer models, upon various stimuli such as hypoxia, but also EGFR signaling and oncogenic signaling through K-Ras G12V and B-Raf V600E, mitochondrial PGK1 phosphorylates pyruvate dehydrogenase (PDH) kinase 1 (PDHK1), subsequently leading to reduced pyruvate utilization within mitochondria through inhibitory phosphorylation of the PDH complex (Li et al., 2016). While this non-canonical kinase activity has not yet been demonstrated in AML, PGK1 expression and glycolytic function have been implicated in AML cell survival and leukemia stem cell biology, suggesting that similar regulatory mechanisms may exist.

### 4.1 Subcellular compartment-specific functions

Compartment-specific functions of glycolytic enzymes are highlighted with the example of glyceraldehyde-3-phosphate dehydrogenase (GAPDH), whose catalytic activity in the cytosol converts glyceraldehyde-3-phosphate into D-glycerate 1,3-bisphosphate. By now, there is a plethora of literature, in various non-hematopoietic systems and cancer models, identifying roles of GAPDH in a very broad landscape of cellular processes such as cell death (Ishitani et al., 1996; Hara et al., 2005), cytoskeletal organization (Kumagai and Sakai, 1983), DNA repair (Meyer-Siegler et al., 1991), tRNA export (Singh and Green, 1993), and membrane fusion and transport (Glaser and Gross, 1995; Tisdale, 2001). The specific activities are thought to be orchestrated by subcellular localization, various protein modifications, and oligomerization.

Among others, GAPDH is a target for oxidative posttranslational modifications such as S-thiolation or S-nitrosylation. Through oxidative modifications of the catalytic cysteine, GAPDH is redirected away from its glycolytic function (Little and O’brien, 1969; Peralta et al., 2015), which leads to a rerouting of carbohydrate flux into the pentose phosphate pathway and the regeneration of cytoplasmic NADPH pools (Ralser et al., 2007). S-nitrosylation of Cys-150 also regulates subcellular distribution as it allows binding between GAPDH and the E3 ubiquitin ligase Siah, after which the complex translocates into the nucleus (Hara et al., 2005). GAPDH acting as a redox sensor was also demonstrated by the fact that attachment of a NAD^+^ irreversibly inhibits GAPDH catalytic activity but leads to binding and activating the transcription factor OCT1 inside the nucleus (Dai et al., 2008). These findings highlight once more cellular metabolic regulation through modification of glycolytic enzymes. For an in-depth discussion of redox regulation of GAPDH we refer the reader to Tossounian et al. (Tossounian et al., 2020).

Of specific interest for AML is the finding that within the nucleus, GAPDH seems to bind to the promyelocytic leukemia protein (PML) and co-localizes with nuclear bodies (Carlile et al., 1998). Disruption of PML nuclear bodies is a distinct feature of acute promyelocytic leukemia (APL) and contributes to apoptosis resistance. However, the impact of GAPDH association with PML and nuclear bodies on disease development and progression is not clear at this point.

Trafficking of GAPDH within the cell could be attributed to microtubule bundling as GAPDH had been shown to increasingly associate with microfilament bundles upon serum starvation (Schmitz and Bereiter-Hahn, 2002). On the other hand, one could also argue that GAPDH might play a more active role in cellular cargo delivery as CHO cells with mutant GAPDH show altered membrane trafficking (Robbins et al., 1995). In a series of publications, Tisdale (2002); Tisdale et al. (2004); Tisdale et al. (2009); Tisdale and Artalejo (2006) established that upon tyrosine phosphorylation, GAPDH is recruited to vesicular tubular clusters and promotes interaction between microtubules and motor proteins, potentially facilitating vesicular trafficking between subcellular compartments. Apart from GAPDH, also phosphoglycerate mutase 1 (PGAM1) has been linked to the organization of cytoskeletal components. In the glycolytic pathway, PGAM1 acts right downstream of GAPDH, converting 3-phosphoglycerate to 2-phosphoglycerate. Independent of its metabolic activity, PGAM1 was reported to directly interact with α-smooth muscle actin, modulating actin filament assembly, cell motility and promoting migration (Zhang et al., 2017; Liu X. et al., 2018). PGAM1 is upregulated in various cancers, and in AML cells Hitosugi et al. showed that PGAM1 supports proliferation in a manner independent of its glycolytic activity (Hitosugi et al., 2012).

Mitochondrial localization of glycolytic enzymes in various cellular systems further illustrates compartment-specific functions. During apoptosis, GAPDH has furthermore been shown to accumulate in mitochondria and promote mitochondrial membrane permeabilization (Tarze et al., 2007). Interestingly, hexokinase 1 (HK1) and HK2 have also been shown to bind to the mitochondrial outer membrane, where they are thought to promote cell survival. Through binding to the voltage-dependent anion channel (VDAC), reviewed in (Pastorino and Hoek, 2008), they restrict access of pro-apoptotic protein Bax to its mitochondrial binding site and therefore inhibit Bax-mediated induction of apoptosis (Pastorino et al., 2002). Another recent report shows that displacement of HK2 from mitochondria-endoplasmic reticulum (ER) contact sites triggered Ca^2+^ release from the ER with subsequent mitochondrial Ca^2+^ overload, resulting in depolarization and cell death (Ciscato et al., 2020), while inducing inflammasome activation in macrophages (Baik et al., 2023). The importance of HK2 association with mitochondria was furthermore established by the finding that HK2 dissociation from mitochondria induced apoptosis, overriding even potent pro-survival growth signals from IGF-1 or insulin (Majewski et al., 2004). Interestingly, a recent report places the anti-apoptotic protein MCL1 at the HK2-VDAC complex at the mitochondria of AML cells concluding that the former stabilizes the complex which not only preserved the outer mitochondrial membrane integrity but also supported HK2 access to mitochondrial ATP allowing cells to escape metabolic stress (Catalano et al., 2023).

The broad landscape of these enzyme activities shows that the function of metabolic enzymes can vary depending on the subcellular distribution, potentially regulated through availability of substrate metabolites or overall metabolic status.

### 4.2 Nuclear functions

Another level of cellular growth control is mediated through transcriptional regulation by phosphofructokinase (PFK), the enzyme catalyzing the third step of glycolysis. When glucose is abundant, nuclear PFK-1 has been shown to bind to the transcriptional cofactors TEADs, which in turn promote the activity of YAP/TAZ, key transcription factors supporting organ growth and cancer cell proliferation (Enzo et al., 2015). On the other hand, Caspi et al. identified aldolase as a positive regulator of Wnt signaling (Caspi et al., 2014). Aldolase, with its isozymes A, B and C, are glycolytic enzymes catalyzing the conversion of fructose 1,6-diphosphate to dihydroxyacetone phosphate and glyceradehyde-3-phosphate. It indirectly promotes Wnt activity inside the nucleus through its cytoplasmic disruption of the inhibitory GSK3β complex (Caspi et al., 2014). Regulation of these important growth pathways through several metabolic players hints at an intricate interplay between the metabolic state of the cell and control of anabolic activity.

Apart from YAP/TAZ and Wnt, another major signaling pathway, Notch, is critically important in hematologic malignancies. Loss of Notch1 mainly affects T-cell development and along this line, Notch1 activating mutations are commonly found in T-cell acute lymphocytic leukemia (Weng et al., 2004). The role of Notch in AML development is less clear, however high levels of different Notch receptors have been reported to be associated with adverse cytogenetic risk and survival in AML (Takam Kamga et al., 2019). Notch1, upon ligand binding, cleaves its intracellular domain to translocate into the nucleus and activate the well-known proto-oncogene c-Myc. This activation can be suppressed through nuclear localization of the glycolytic enzyme α-enolase (ENO1), which converts 2-phosphoglycerate to phosphoenolpyruvate in glycolysis (Hsu et al., 2008). The close relationship between enolase and c-MYC regulation is furthermore highlighted by the previously mentioned alternative transcript of ENO1, MBP1, whose nuclear localization again directly lowers c-Myc levels (Subramanian and Miller, 2000).

An interesting report by Thomas G.E. et al. highlighted the nuclear localization of HK2 in AML cells and linked it to stemness properties. This function was confined to nuclear HK2 and independent of its kinase function. While the exact mechanism of action remains unclear, the study suggests that HK2 likely influences chromatin accessibility through chromatin remodeling proteins, impacts the DNA damage response via interactions with DNA damage response proteins, and regulates transcription by interacting with transcriptional regulatory proteins (Thomas et al., 2022). In our own report on HK3, an isozyme of HK2, we discovered that it also localizes to the nucleus in AML cells, however, whether or how this affects its catalytic function remains to be investigated (Seiler et al., 2022).

Another interesting example is Triosephosphate isomerase (TPI), catalyzing the fourth step of glycolysis, the reversible interconversion of dihydroxyacetone phosphate (DHAP) and glyceraldehyde-3-phosphate (G3P). TPI nuclear localization was found to play a role in promoting tumor migration, colony formation and confers chemotherapy resistance, independent of its enzymatic activity in lung adenocarcinoma (Liu et al., 2022).

Regulation of transcriptional activity through epigenetic modification is another area, where glycolytic enzymes have shown involvement. As mentioned previously, PKM2 affects transcription through histone phosphorylation in the nucleus (Yang et al., 2012a). In cervical cancer, LDHA was shown to translocate into the nucleus because of ROS induction. Once in the nucleus, LDHA converts α-ketobutyrate into α-hydroxybutyrate, which in turn leads to H3K79 hypermethylation (Liu Y. et al., 2018). Consequently, antioxidant genes are upregulated along with Wnt target genes, resulting in tumor progression (Liu Y. et al., 2018).

There is a multitude of other metabolic enzymes that have recently been identified to play roles in the tight regulation of key cellular processes such as proliferation and differentiation. Gluconeogenic enzyme fructose 1,6-bisphosphatase (FBP-1), TCA cycle enzymes PDH and malate dehydrogenase (MDH1), or G6PD are a few examples of various metabolic enzymes regulating major processes such as transcription and RNA binding, apart from their metabolic activity (Cieśla, 2006; Chueh et al., 2011; Li et al., 2014; Shamloo et al., 2024).

### 4.3 Glycolytic enzymes as regulators of autophagy

Autophagy is a recycling and catabolic pathway, through which cells degrade cytosolic material. Ranging from degradation of unspecific material such as old or damaged organelles to clearing of specific proteins based on target sequence recognition, autophagy is crucial for the maintenance of cellular integrity and health. Autophagy levels are high in HSCs and, through clearance and recycling of components such as mitochondria, protect them from metabolic stress (Warr et al., 2013; Ho et al., 2017). As most HSCs are quiescent, they cannot readily dilute harmful cytosolic material to be passaged to daughter cells [reviewed in (Riffelmacher and Simon, 2017)]. HSCs are thought to have low metabolic activity, and autophagy might be supporting energetic needs through recovery and recycling of building blocks from various sources. Loss of autophagy in HSCs causes the accumulation of mitochondria and induces accelerated myeloid differentiation, underlining the importance of a tight autophagy regulation within the HSC compartment (Ho et al., 2017). While generally autophagy is thought to be tumor suppressive through maintenance of cellular health, the advantageous and protective attributes of autophagy induction can also be harnessed by cancer cells, prominently shown in CML long-term LSCs which survive in hypoxic or hostile environments (Baquero et al., 2019). For a detailed review on the interplay between autophagy and AML metabolism we recommend the following resource (Chen et al., 2024).

There are multiple levels on which glycolytic enzymes regulate autophagic activity. Autophagy is activated through starvation-induced translocation of GAPDH into the nucleus. Under glucose starvation, AMP-activated protein kinase (AMPK) phosphorylates GAPDH, leading to its nuclear translocation, where it promotes Sirt1 activity (Chang C. et al., 2015), which in turn directly correlates with autophagic activity (Hariharan Nirmala et al., 2010). Furthermore, PGK1, a protein kinase, phosphorylates Beclin-1 (BECN1), a regulatory subunit of the early stage autophagy complex PI3K-III (Qian et al., 2017). Initiation of autophagy requires the assembly of multiprotein complexes, such as ULK1 complex and PI3K-III complex, to bring about autophagosome nucleation and autophagosome formation [reviewed in (Hurley and Young, 2017)]. Under hypoxic conditions, PGK1 mediated phosphorylation of BECN1 at S30 promotes recruitment and activity of another complex component, vacuolar sorting protein-34 (VPS34), leading to an increase in autophagy activity (Qian et al., 2017). In AML, PKM2 has also been shown to increase the phosphorylation of BECN1 along with autophagy activation (Wang et al., 2019). Apart from PGK1 and PKM2, acting as a metabolite sensor, GAPDH exerts inhibitory binding of mTOR activator Rheb under low glucose conditions (Lee et al., 2009). The resulting inhibition of mTORC1 leads to an induction of autophagy, since mTORC1 and its proliferative signaling cascade are among the most potent inhibitors of catabolic pathways, including autophagy. GAPDH therefore translates glycolytic flux to the major metabolic regulator mTORC1. HK2 has also been shown to directly bind to and inhibit mTORC1 when glucose levels are low, pointing to a multi-level glycolytic regulation over mTORC1 activity (Roberts et al., 2014).

## 5 Targeting glycolytic enzymes in AML therapy

**FIGURE 6:**
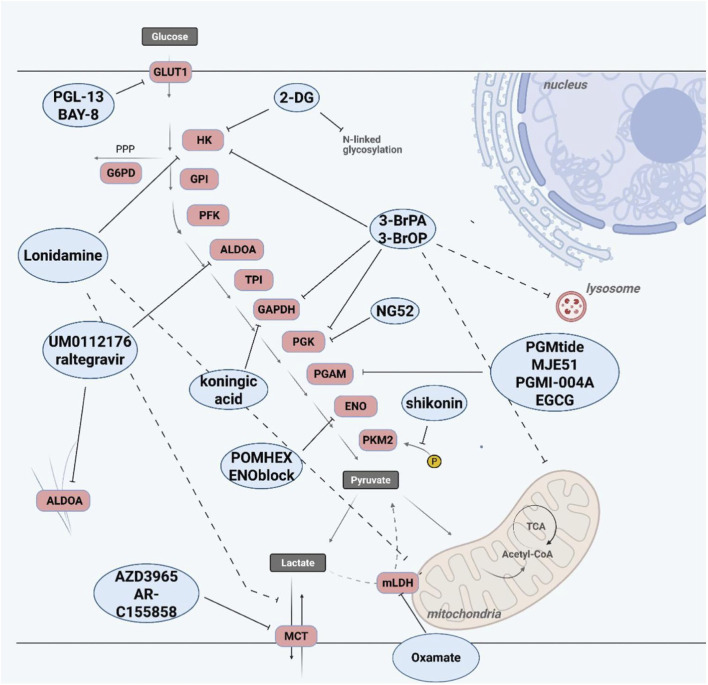
Pharmacological targeting of glycolysis and associated metabolic pathways. Schematic overview of small-molecule inhibitors targeting key steps of glycolysis and related metabolic processes. Glucose uptake can be inhibited at the level of GLUT1 (e.g., PGL-13, BAY-876), thereby limiting substrate availability. Upstream glycolytic enzymes are targeted by compounds such as 2-deoxy-D-glucose (2-DG), which inhibits hexokinase activity and interferes with N-linked glycosylation, and lonidamine, which affects hexokinase and mitochondrial function. Broad-spectrum inhibitors such as 3-bromopyruvate (3-BrPA) and its derivative 3-BrOP target multiple glycolytic enzymes. Further downstream, GAPDH can be inhibited by koningic acid, while enolase inhibitors (e.g., POMHEX, ENOblock) and pyruvate kinase modulators (e.g., shikonin) disrupt late glycolytic steps. Aldolase activity can be targeted by UM0112176 and raltegravir. In addition, PGAM inhibitors (e.g., PGMI-004A, MJE51, PGMTide, EGCG) interfere with intermediate steps of glycolysis. Beyond glycolysis, metabolic targeting extends to lactate production and transport. Lactate dehydrogenase (LDH) inhibitors (e.g., oxamate) and monocarboxylate transporter (MCT) inhibitors (e.g., AZD3965, AR-C155858) disrupt lactate metabolism and export, thereby affecting tumor microenvironment and metabolic homeostasis. Collectively, these compounds highlight multiple actionable metabolic vulnerabilities in cancer cells, including acute myeloid leukemia.

An overview of drugs targeting metabolism in AML is shown in Figure 6 and Table 2.

**TABLE 2:** Overview of compounds targeting components of the glycolytic pathway.

Compound	Primary target	Stage of development	Key reference/Source
*Upper glycolytic pathway*			
2-DG	HK	preclinical for combination therapies	Pajak et al. (2019)
3-BrPA/3-BrOP	HK2, GAPDH, 3-PGK, mitochondrial, lysosomal and redox metabolism	3-BrOP: preclinical	Ganapathy-Kanniappan et al. (2009), Pereira da Silva et al. (2009), Akers et al. (2011), Fan et al. (2019)
Lonidamine	HK, glycolysis, mitochondrial respiration (complex II), lactic acid transport	Production discontinued	Nath et al. (2013), 2016
UM0112176	Aldolase A	preclinical	Gizak et al. (2019)
Raltegravir	Aldolase A	preclinical	Chang et al. (2019)
Koningic acid	GAPDH, DNA polymerase β and λ	preclinical	Rahier et al. (2015)
PGL-13	GLUT1	preclinical	Reckzeh and Waldmann (2020)
BAY-876	GLUT1	preclinical	Miller et al. (2024)
*Lower glycolytic pathway*			
NG52	PGK1	preclinical	Wang et al. (2021)
PGMtide	PGAM	preclinical	Engel et al. (2004)
MJE51	PGAM	preclinical	Evans et al. (2005)
PGMI-004A	PGAM	Preclinical	Hitosugi et al. (2012)
EGCG	PGAM	Preclinical	Li et al. (2017)
Enoblock	ENO1	Preclinical	Cho et al. (2017)
POMHEX	ENO1	Preclinical	Lin et al. (2020)
Shikonin	PKM	Preclinical	Zhao et al. (2018)
AZD3965	MCT1	Clinical, phase I	Guan and Morris (2020)
AR-C155858	MCT1	Preclinical	Saulle et al. (2021)

### 5.1 Inhibitors targeting the upper glycolytic pathway

#### 5.1.1 Hexokinase inhibitors

*2-Deoxy-D-glucose (2-DG)* is one of the earliest compounds used to target glucose metabolism. As a glucose analog, it competitively inhibits glycolysis. After phosphorylation by hexokinase, 2-DG-6-phosphate cannot be further metabolized, leading to its accumulation. This accumulation allosterically inhibits hexokinase activity and reduces its association with mitochondria (Lynch et al., 1991).

*In vitro*, 2-DG inhibits glycolysis, depletes intracellular ATP levels, and reduces cell proliferation. However, relatively high doses are required to induce apoptosis. Its efficacy also depends on glucose availability and the ability of cancer cells to switch to oxidative phosphorylation (OxPHOS) for ATP generation. Despite promising preclinical data, clinical translation has been limited. Early-phase clinical trials using 2-DG as a single agent or in combination therapies for solid tumors reported modest efficacy and insufficient responses (Pajak et al., 2019). Furthermore, 2-DG lacks tumor selectivity. Tumor-infiltrating immune cells often exhibit higher glucose uptake than cancer cells, which may impair antitumor immunity upon treatment (Reinfeld et al., 2021). These limitations, together with the requirement for high doses, raise concerns about its therapeutic potential as a single agent.

Recent studies suggest that 2-DG may be more effective in combination strategies. In FLT3-ITD mutated AML, 2-DG has shown promising *in vitro* activity (Larrue et al., 2015; Ju et al., 2017). Notably, its antileukemic effects appear to be largely independent of glycolysis inhibition. In addition to blocking glycolysis, 2-DG interferes with N-linked glycosylation, leading to altered protein expression and signaling (Andresen et al., 2012; Mühlenberg et al., 2015). In FLT3-ITD AML, inhibition of glycosylation reduces oncogenic signaling. Consistently, mannose supplementation restores glycosylation and rescues cell growth, but does not reverse ATP depletion (Larrue et al., 2015). This indicates that disruption of glycosylation, rather than ATP depletion, is the dominant mechanism underlying its cytotoxic effects.

*3-Bromopyruvate (3-BrPA)* is a pyruvate mimetic with broad effects on cellular metabolism. It exerts antineoplastic activity primarily through alkylation of thiol groups in cysteine residues. As a result, it inhibits multiple glycolytic enzymes, including hexokinase 2 (HK2), GAPDH, and phosphoglycerate kinase, as well as mitochondrial and redox-related enzymes (Ganapathy-Kanniappan et al., 2009; Pereira da Silva et al., 2009; Fan et al., 2019).

However, clinical development has been challenging. Unformulated 3-BrPA causes pain upon intravenous administration, is rapidly inactivated in serum, and shows limited retention in tumor cells (Fan et al., 2019). These pharmacokinetic and toxicity issues have hindered its clinical translation. A related compound, 3-bromo-2-oxopropionate-1-propyl ester (3-BrOP), appears to overcome some of these limitations. *In vitro* studies demonstrated sensitivity in all tested AML cell lines (Akers et al., 2011). However, *in vivo* validation is currently lacking, and no clinical trials have been listed to date.

*Lonidamine* is a derivative of indazole-3-carboxylic acid with pleiotropic effects on cellular metabolism. Although often described as a hexokinase inhibitor, it also affects mitochondrial respiration and lactate transport. In Ehrlich ascites tumor cells, lonidamine inhibited mitochondrially bound hexokinase activity by up to 68% (Floridi et al., 1981). In addition, lonidamine inhibits mitochondrial respiratory chain complex II, increases intracellular reactive oxygen species (ROS), and interferes with monocarboxylate transporter–mediated lactate export (Nath et al., 2016). The relative contribution of these mechanisms to its anticancer activity remains unclear. Clinically, lonidamine has shown modest efficacy as a single agent but favorable tolerability. Its primary clinical value appears to lie in combination therapies, where it enhances the effects of chemotherapeutic agents (Di Cosimo et al., 2003; Nath et al., 2013). Current research focuses on more potent derivatives and optimized combination strategies rather than late-phase clinical development (Cervantes-Madrid et al., 2015; Huang et al., 2020).

#### 5.1.2 GAPDH inhibitors

Glyceraldehyde-3-phosphate dehydrogenase (GAPDH) is an attractive target due to its role in regulating glycolytic flux, particularly in cancer cells. In addition to modification by endogenous metabolites, GAPDH can be inhibited by small molecules such as koningic acid (KA) (Liberti et al., 2017). KA has shown cytotoxic effects *in vitro* and tumor-selective activity in a breast cancer mouse model (Rahier et al., 2015; Liberti et al., 2017). However, other *in vivo* studies have failed to demonstrate significant therapeutic benefit (Rahier et al., 2015), highlighting variability in response. More recently, a novel inhibitor, spirocyclic 3-bromo-4,5-dihydroisoxazole (BDHI), was shown to form irreversible covalent bonds with the catalytic cysteine of GAPDH (Galbiati et al., 2023). This suggests improved potency compared to KA. However, further preclinical and clinical validation in AML treatment is required.

#### 5.1.3 Glucose uptake inhibition

An alternative strategy to targeting glycolytic enzymes is to inhibit glucose uptake at the level of transmembrane glucose transporters (GLUTs). Several potent GLUT inhibitors have been developed, targeting either specific isoforms or multiple transporters (Reckzeh and Waldmann, 2020). Preclinical studies have yielded mixed results, although some compounds demonstrate significant tumor growth inhibition in solid cancers. In AML, GLUT1 has emerged as a critical metabolic dependency. Genetic ablation of GLUT1 impairs AML cell proliferation (Saito et al., 2015). Pharmacological inhibition of GLUT1 has also shown promise. The GLUT1 inhibitor PGL-13 enhances the cytotoxic effects of cytarabine (Ara-C) and doxorubicin in AML cell lines with high GLUT1 expression (Åbacka et al., 2021). In addition, a CRISPR screen identified GLUT1 as essential for leukemia stem cell survival in a murine MLL:AF9 AML model. GLUT1 inhibition suppressed glycolysis, induced autophagy and apoptosis, and promoted differentiation, resulting in improved survival *in vivo*. Combination strategies appear particularly promising. Dual inhibition of GLUT1 and oxidative phosphorylation impaired metabolic plasticity in primary AML samples, especially in RUNX1-mutated cells, while sparing healthy cells (Rodriguez-Zabala et al., 2023). Despite strong preclinical evidence, no GLUT1 inhibitors have yet advanced to clinical trials. However, newer compounds such as BAY-876 show potent inhibition of glucose uptake and induction of apoptosis in preclinical models (Miller et al., 2024).

### 5.2 Inhibitors targeting the lower glycolytic pathway

#### 5.2.1 PGK inhibitor

NG52 is a preclinical tool compound inhibiting yeast cyclin-dependent kinase reported to inhibit PGK1 kinase activity. It has not entered clinical trials. NG-52 has demonstrated promising anti-cancer activity by inhibiting tumor cell proliferation, inducing apoptosis, and altering cancer cell metabolism in models of glioma and ovarian cancer (Gou et al., 2021; Wang et al., 2021).

#### 5.2.2 PGAM inhibitors

Inhibition of PGAM through either a synthetic cell-penetrating peptide PGMtide (Engel et al., 2004) or small molecule compounds such as MJE51 (Evans et al., 2005) or PGMI-004A (Hitosugi et al., 2012) was shown to induce cancer cell growth arrest *in vitro*. In AML, PGMI-004A, derived from the known PGAM1 inhibitor Alizarin Red S, was shown to reduce lactate production and hamper cell line and primary leukemia proliferation, while causing minimal toxicity to normal blood cells (Hitosugi et al., 2012). Interestingly, a recent report showed that (−)- Epigallocatechin-3-gallate (EGCG), a natural catechin of green tea extract, attenuated glycolysis and cancer cell proliferation through PGAM1 inhibition (Li et al., 2017). In a leukemia specific context, EGCG has also been shown to enhance *in vitro* differentiation therapy of certain AML subtypes and a more detailed review on the other effects of green tea catechins in hematological malignancies can be viewed here (Mokra et al., 2022; Della Via et al., 2023).

#### 5.2.3 Enolase inhibitors

A small molecule enolase inhibitor, POMHEX, has been identified in the context of glioma, which often have a collateral deletion of ENO1 and therefore depend on the paralogue ENO2 (Lin et al., 2020). Another enolase inhibitor, ENO block, has been reported mostly in the context of type 2 diabetes mellitus, where it was shown to reduce hyperglycemia and increase enolase nuclear translocation (Cho et al., 2017). It has recently been shown to reduce *in vitro* and *in vivo* colorectal cancer cell growth (Hu et al., 2020).

#### 5.2.4 PKM inhibitors

Shikonin, a natural product isolated from the roots of certain herbs, has been attributed very broad anti-inflammatory, anti-oxidant and anti-cancer effects [reviewed in (Guo et al., 2019)]. Interestingly, one mechanism through which shikonin was shown to inhibit aerobic glycolysis in lung and melanoma cell line models is via decreasing PKM2 phosphorylation (Zhao et al., 2018).

#### 5.2.5 MCT inhibitors

Monocarboxylate transporters (MCTs) shuttle lactate across the cell membrane, mediating both release as well as import of lactate. While inhibiting MCTs leads to the intracellular accumulation of lactate, it also disrupts the metabolic fuel supply by cutting off the lactate import required for oxidative energy metabolism in cancer cells. This is particularly relevant in AML, where OXPHOS-reliant cells utilize MCT1 and mitochondrial-associated LDH to increase lactate-derived pyruvate flux into the TCA cycle, thereby sustaining mitochondrial respiration. Consequently, pharmacological inhibition of MCT1, specifically using AZD3965 (Monteith et al., 2024) or its analogue AR-C155858 (Saulle et al., 2021), has been shown to inhibit leukemia cell proliferation and potentiate the cytotoxicity of standard treatments like Ara-C (Saulle et al., 2021) or BET inhibition (Monteith et al., 2024). AZD3965 has recently shown promise in clinical trials (NCT01791595), establishing a recommended Phase 2 dose for tumors expressing high MCT1 and low MCT4 (Halford et al., 2023). However, emerging evidence suggests that dual MCT1/MCT4 blockade may be necessary for broader efficacy in AML, as MCT4 upregulation further supports leukemia growth and the maintenance of leukemia-initiating cells.

#### 5.2.6 LDH inhibitors

LDH regenerates NAD^+^ to sustain glycolytic flux and serves as a critical bridge between glycolysis and mitochondrial metabolism. Although selective LDH inhibitors have yet to transition into routine clinical use for AML, preclinical studies using the broad-spectrum inhibitor oxamate have highlighted lactate metabolism as a therapeutic vulnerability. Specifically, oxamate reduces mitochondrial pyruvate production by inhibiting mitochondrial LDH, which facilitates the conversion of lactate into pyruvate, thereby disrupting the TCA cycle. This metabolic blockade has been shown to sensitize AML cells to BET inhibition both *in vitro* and *in vivo* (Monteith et al., 2024).

### 5.3 Resistance mechanisms and combination therapies

With any cancer treatment, development of drug resistance is a massive challenge and metabolic plasticity of cancer cells is most certainly a major concern in refractory/residual disease. A report by Jones et al. exemplifies metabolic adaption upon treatment. During relapse, LSC population pretreated with venetoclax and azacytidine, a combination that targets amino acid (especially cyst(e)ine uptake and utilization to support TCA cycle and electron transport chain (ETC) activities), upregulates fatty acid oxidation to compensate for their metabolic impairments due to OxPHOS inhibition during pretreatment (Jones et al., 2018). However, it is not entirely clear whether cancer cells are generally more plastic than their normal counterparts. On the other hand, certain aspects of cellular energy metabolism have been shown to play a key role in the development of resistance against commonly used therapies, such as enhanced oxidative metabolism in either AraC-resistant AML cells or IDH mutant AML cells resistant to specific IDH inhibitors (Farge et al., 2017; Stuani et al., 2021). Therefore, energy metabolism reprogramming compounds could prove highly valuable in combination therapy regimens. For example, combination of the BCL-2 inhibitor venetoclax with glycolysis inhibitor 2-DG has been shown to efficiently kill various cancer cells, including AML, *in vitro* whereas single treatments of each compound only partially induced apoptosis (Yamaguchi et al., 2011; Grønningsæter et al., 2020). Furthermore, additional metabolism-targeting therapies have shown to potentiate the effects of venetoclax in treating AML, such as 2-chloro-adenosine, a potent inhibitor of mitochondrial ATP synthase (Buettner et al., 2021). Recent studies demonstrated that therapy-resistant AML cells can compensate for glycolytic inhibition by increasing lactate uptake through MCT1 and mitochondrial-associated LDH to sustain TCA cycle activity and OXPHOS. Pharmacologic targeting of this lactate-fueled metabolic bypass sensitized AML cells to BET inhibition (Monteith et al., 2024). Similarly, inhibition of the TCA cycle enzyme α-ketoglutarate dehydrogenase by the imipridone ONC-213 induced mitochondrial stress responses and reduced MCL1 expression, revealing additional vulnerabilities within OXPHOS-dependent leukemic cells (Su et al., 2024). These complementary metabolic adaptations and therapeutic implications are comprehensively discussed in a recent commentary (Boët and Sarry, 2024). These combination treatments are promising approaches to combat therapy resistance and target metabolically heterogeneous cancer cell populations.

### 5.4 Responses of healthy immune cells to glycolysis-targeting therapies

When broadly targeting cellular energy metabolism, the question arises to what extent the antitumoral defense will be affected. As established before, certain metabolic features are shared between activated immune cells and cancer cells, such as the upregulation of genes involved in glycolysis and glutaminolysis as well as increased flux through glycolytic pathways (Frauwirth et al., 2002; Wang et al., 2011). Interestingly, a recent report showed quite stunning results quantifying the uptake of labeled FDG within different cell populations within the tumor microenvironment of selected solid tumors (renal, breast and colon cancer). FDG has been used in diagnostic cancer imaging for a long time to visualize tissue with enhanced glucose uptake. Surprisingly, tumor-infiltrating immune cells, specifically myeloid cells, had a roughly 3 times higher FDG uptake compared to cancer cells (Reinfeld et al., 2021). Furthermore, targeting energy metabolism might affect differentiation and/or activation status of immune cells, with the theoretical potential for both enhancing or repressing immune response. Using a mouse melanoma spheroid model as well as an *in vivo* mouse lymphoma model, it was shown that transient glucose restriction in fact enhanced tumor-specific CD8^+^ T-cell effector function and tumor clearing (Klein Geltink et al., 2020).

Another important aspect is highlighted in a recent review discussing the effects of lactate on immune cells and how lactate can be targeted and utilized in a context-dependent manner (Li and Cui, 2023; Mortazavi Farsani and Verma, 2023).

### 5.5 Lack of specific glycolytic inhibitors

Since we have entered the era of precision medicine and targeted therapies, there is a need for developing compounds that specifically target components of the glycolytic pathway. Given the advancing understanding of certain glycolytic enzymes supporting proliferation not only through their metabolic activity but also through growth promoting regulatory functions, their therapeutic exploitation should be investigated. As pointed out earlier, multiple isoforms of glycolytic enzymes are associated with malignant development and their upregulation in cancer cells could potentially serve as a point of attack. Among these are HK2, PKM2 and LDHA, for which development of selective inhibitors is underway (Lin et al., 2016; Kim et al., 2019). Another approach under investigation is specific targeting of important regulators of glycolytic enzymes, such as HIF-1 or MYC, two major activators of glycolytic metabolism active in many cancers.

## 6 Challenges of metabolic research

The cellular metabolic landscape is enormously complex and understanding how the different pathways function together remains challenging. Furthermore, progress within the field might have been hampered due to the difficulty to mimic *in vivo* conditions with current experimental conditions. We are still learning how significant the impact of readily used culture conditions is on the *in vitro* metabolic profile.

For culturing blood cells, RPMI1640 is widely used; this medium was developed in the 1960s with the primary goal of supporting robust cellular growth *in vitro*. As a result, many of its components and nutrients are present at concentrations far exceeding those found in human plasma. While this approach ensures cell survival and proliferation, it has contributed to a disconnect between *in vitro* findings and *in vivo* outcomes, often leading to disappointing translational results. A notable example is the role of glutamine in fueling the TCA cycle in lung cancer and glioblastoma: while *in vitro* studies using standard media suggest a strong dependence on glutamine as a carbon source, *in vivo* isotope tracing has revealed that glutamine contributes minimally to the TCA cycle in these tumors. Instead, lung cancers *in vivo* predominantly utilize the glucose-pyruvate pathway, with pyruvate carboxylase facilitating the entry of glucose-derived carbons into the TCA cycle (Davidson et al., 2016). It remains an open question whether this discrepancy is primarily due to the supraphysiological glutamine and nutrient levels in conventional media, and whether it can be resolved by using media that more closely mirror human plasma composition. Recent advances in human plasma-like medium (HPLM) have enabled researchers to culture cells in conditions that closely mimic the metabolite composition of human plasma, leading to more physiologically relevant metabolic activity and drug responses compared to traditional media (Cantor et al., 2017; Voorde et al., 2019; Ceranski et al., 2025). Studies have shown that HPLM can uncover metabolic dependencies and drug sensitivities that are not observed under standard culture conditions, thereby enhancing the translational relevance of *in vitro* experiments in cancer and metabolic research (see comprehensive reviews (Golikov et al., 2022; Hammond et al., 2024)). HPLM is now commercially available, supports a wide range of cell types, and is increasingly used in high-throughput drug screening and metabolic studies. On a more encouraging note, the fact that distinct metabolic phenotypes within cell populations can be identified even *in vitro* culture conditions shows that we are potentially capturing important *in vivo* cellular features.

Another challenge in metabolic research was recently highlighted in an extensive review on metabolic heterogeneity in cancer by Kim and DeBerardinis (Kim and DeBerardinis, 2019), where they mention the need to investigate combinatorial effects of co-occurrent mutations. Metabolic effects of oncogenic drivers have largely been investigated through the insertion of single mutations into cellular and/or mouse models, while cancers harbor a much more extensive mutational background which potentially influences the metabolic effects greatly. The same holds true for characterization of glycolytic enzymes, where it can be tricky to directly attribute certain features to specific enzymes when loss of the investigated enzyme elicits broad changes in various cellular metabolic pathways. Furthermore, many AML studies are performed using immunodeficient mouse models, limiting insight into how metabolic interventions impact antitumoral immune responses - an important consideration, as metabolism shapes immune cell activation and differentiation. The emergence of spatial metabolomics promises to transform this landscape, enabling the simultaneous mapping of metabolic activity and cellular context within the tumor microenvironment. This approach may help disentangle cell-intrinsic and microenvironment-driven metabolic programs and clarify how therapies affect both malignant and immune compartments. A recent bibliometric review highlights a surge in spatial metabolomics research in cancer, particularly in immuno-oncology and metabolic reprogramming, underscoring its growing importance in translational research (Chen et al., 2025).

## 7 Concluding remarks

Increasing knowledge on how glycolysis and glycolytic enzymes regulate metabolic dynamics, cellular fate, proliferation, stem cell function, and cancer initiation, as well as their role in developing treatment resistance mechanisms, should help to identify suitable cancer therapy targets. While there is ample evidence showing that various glycolytic enzymes are active in multiple pathways apart from glycolysis, their regulatory functions in an AML-specific context are not yet understood. Another layer of complexity regarding therapeutic opportunities is added by the role of dietary and host metabolism influences on cancer growth and progression. Even though these questions are beyond the scope of this review, we find it important to mention this emerging field of investigation (Lien and Vander Heiden, 2019). More research shedding light on the effects of dietary regimens on regulation and activity of metabolic enzymes in the context of cancer will surely provide exciting new insights. Many metabolic enzymes have compartment-specific, including nuclear, functions and their regulation of (trans)location requires further investigation. However, understanding these regulatory functions could prove to be crucially important in tackling cancer. Glycolysis is certainly not the only cellular pathway for energy generation (OxPHOS, fatty acid oxidation), but targeting growth regulatory enzymes that are also involved in metabolite conversion potentially harbors the advantage of disrupting two pathways at once and may prove especially effective in combinatory treatments with other cytotoxic or targeted agents. These combination regimens represent a very promising avenue to enhance therapeutic options for AML patients and hopefully minimize relapse by providing the option of targeting heterogeneous tumor populations.
